# Increased Fetal Thymocytes Apoptosis Contributes to Prenatal Nicotine Exposure-induced Th1/Th2 Imbalance in Male Offspring Mice

**DOI:** 10.1038/srep39013

**Published:** 2016-12-15

**Authors:** Ting Chen, You-e Yan, Sha Liu, Han-xiao Liu, Hui-yi Yan, Li-fang Hou, Wen Qu, Jie Ping

**Affiliations:** 1 Department of Pharmacology, Wuhan University School of Basic Medical Sciences, Wuhan 430071, China

## Abstract

Nicotine, a definite risk factor during pregnancy, is an immunomodulator. This study was designed to investigate the effects of prenatal nicotine exposure (PNE) on the balance of Th1/Th2 in offspring, and further explore the developmental origin mechanisms from the perspective of fetal thymocytes apoptosis. Pregnant Balb/c mice were administered 1.5 mg/kg nicotine subcutaneously twice per day from gestational day (GD) 9 to GD18. Results showed that PNE could cause a Th2 shift in male offspring, manifested as increased ratio of IgG1/IgG2a, IL-4 production in serum, and IL-4/IFN-γ expression ratio in spleen. Increased apoptosis of total thymocytes and CD4SP and reduced cell proportion of CD4SP were found in PNE male offspring on postnatal day (PND) 14 and PND 49. In the fetuses, decreased body weight and organ index of fetal thymus, histological changes in fetal thymus, reduced CD4SP proportion and increased fetal thymocyte apoptosis were observed in nicotine group. The increased mRNA expression of genes involved in Fas-mediated apoptotic pathway and protein expression of Fas were also detected. In conclusion, PNE could cause a Th2 shift in male offspring mediated by reduced CD4^+^ T cells output, which may result from the increasing apoptosis of total thymocytes and CD4SP.

Epidemiological studies showed that immune and inflammatory disorders had become a growing health problem worldwide^1^,^2^. In recent years, increasing evidence suggest that immune and inflammatory disorders may develop from in utero insults^3^. The exposure to adverse intrauterine environment had been shown to be associated with some immune diseases in offspring after birth, such as asthma. Prenatal tobacco smoke exposure is a definite risk factor for adverse intrauterine environment. During pregnancy, approximately 25~29% of pregnant women continued smoking and about half of reproductive-aged women were exposed to second-hand smoke^4^,^5^. Nicotine, the major alkaloid of tobacco, is thought to be the main toxic component harming health^6^. In addition, nicotine can easily cross the placental barrier because of its low molecular weight and high lipid solubility^7^. Both epidemiological and experimental animal studies showed that the increased risks of adult immune diseases was related to prenatal tobacco smoke or nicotine exposure^8^,^9^. Therefore, prenatal nicotine exposure (PNE) is one of the risk factors for developmental origins of immune diseases.

The balance of helper T cell 1(Th1)/Th2 is necessary to maintain the immune homeostasis. The disruption of this balance is suggested to be one of the potential mechanisms of the immune dysfunction^10^. The body is prone to autoimmune diseases when there was a Th1 shift or increased Th1 cells secreting interferon-γ (IFN-γ)^11^,^12^, and allergic diseases were susceptible due to a Th2 skewing. Studies showed that a Th2 skewing enhanced the susceptibility of airway inflammation, bladder cancer or colorectal cancer and so on^13^,^14^,^15^. Moreover, it was reported that tobacco smoke and nicotine exposure during prenatal and postnatal life could alter the immune responses and cause a Th2 shift, which could increase the prevalence of wheezing during childhood^16^. Therefore, the imbalance of Th1/Th2 induced by PNE is closely related to the susceptibility to immune diseases in offspring. However, the underlying mechanism responsible for the intrauterine programming of Th1/Th2 skewing remains unclear.

The development of Th1 and Th2 were originated from thymocytes. The thymus is the earliest developed immune organ in fetus, and it is the primary site of T cell production. The normal development of thymus is essential to establish the competent immune function during the fetal and postnatal stage. Based on the expression of cell surface markers CD4 and CD8, the differentiation progress of thymocytes can be divided into a series of stages: immature CD4^−^CD8^−^ double negative cells (DN) differentiate into CD4^+^CD8^+^ double positive cells (DP), which then give rise to single positive cells (SP) that only express CD4 or CD8^17^. After migrating out of the thymus, the mature native T cells differentiate into effector T cells (such as Th) under the stimulation of cytokines in periphery. Several studies showed that the abnormal development of the thymus, such as reduced output or changed proportion of phenotypes, could lead to the dysfunction of peripheral effector T cells^18^,^19^,^20^. Owing to the high lipid solubility, nicotine can be easily absorbed and accumulated in the fetus after crossing the placental barrier. The thymus is one of the toxic target organs of nicotine, and the development of thymocytes might be disturbed by nicotine. By using the fetal thymus organ culture (FTOC), researchers found that nicotine could increase immature thymocytes and decrease the mature thymocytes^21^. Furthermore, nicotine also could hamper the adhesion and growth of thymic epithelial cells^22^. Previously we had confirmed that tobacco smoke or nicotine exposure during middle and late pregnancy could lead to intrauterine growth retardation (IUGR)^23^. Lots of studies suggested that IUGR was accompanied by thymic hypoplasia, such as decreased total number of thymocytes and percentages of CD4^+^SP and CD8^+^SP^24^. Thus we speculated that fetal thymic hypoplasia induced by PNE might be involved in mediating the abnormal immune responses of peripheral effector T cells in offspring.

Apoptosis is crucial during the development process of thymocytes. Increased apoptosis of thymocytes in fetal stage could reduce the output and change the phenotypes of thymocytes. Nicotine is an activator for nicotinic acetylcholine receptor (nAChR), which might regulate the development of thymocytes. Fetal thymocytes express multiple nAChRs. Studies showed that T cell nicotinic responsiveness occurred through α7 subunit^25^. Researchers found nicotine interacted with α7 nAChR, which might mimic TCR signals. And the thymocytes would be lost due to their lack of a TCR specific for self-MHC. So the interaction between nicotine and α7 nAChR could induce the apoptosis of thymocytes^21^. Fas was involved in the apoptotic signal pathway in T cells. In addition, nicotine could trigger an influx of extracellular Ca^2+^ following α7 nAChR stimulation, which could activate the signaling pathway involved in Fas^21^. Therefore, we speculated that nicotine might impair the development of fetal thymocytes by inducing the thymocyte apoptosis through α7 nAChR/Fas pathway.

In this study, we investigated the effects of PNE on the development of fetal thymocytes and the balance of Th1/Th2 in male offspring after birth, and further explored the possible developmental origin mechanisms by detecting the thymocytes apoptosis and the gene expressions of apoptotic pathway in fetal thymus. The present study will provide evidence for elucidating the developmental toxicity of nicotine on fetal thymus and be conducive to better understand the developmental origin of immune dysfunction in nicotine-exposed offspring.

## Results

### Results of offspring after birth

#### Developmental parameters of male offspring

**Figure 1:**
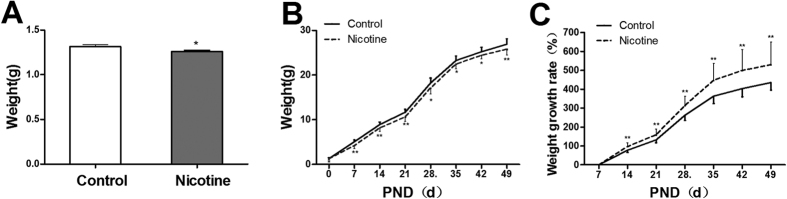
Effects of prenatal nicotine exposure (PNE) from gestational day (GD) 9 to GD18 on body weight at postnatal day 0 (PND0), body weight and growth rate of male offspring from PND0 toPND49. (**A**) Body weight at PND0; (**B**) Changes of body weight in male offspring mice; (**C**) Body weight growth rate in male offspring mice. Mean ± SD, n = 20. ^*^P < 0.05, ^**^P < 0.01 vs control.

Body weight is an important index for development. The results showed that the body weights of male offspring in PNE group were lower than those of controls from postnatal day (PND)0 to PND49 (*P* < 0.01, *P* < 0.05, Fig. 1A,B), but the growth rates were higher compared with the control (*P* < 0.01, Fig. 1C).

#### Levels of IgG1, IgG2a and interleukin (IL)-4 in serum

**Figure 2:**
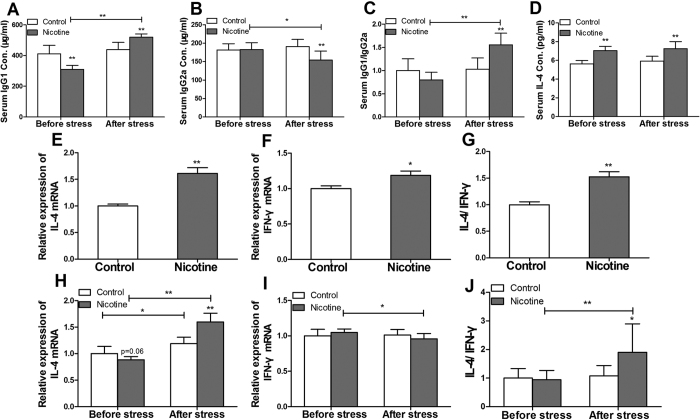
Effects of prenatal nicotine exposure (PNE) on the levels of IgG1, IgG2a, the ratio of IgG1/IgG2a, interleukin-4 (IL-4) in serum after S. pneumoniae immunization on PND49 and the mRNA expression of IL-4, interferon-γ (IFN-γ), expression ratio of IL-4/IFN-γ in the spleen in the male offspring on PND14 and PND49 after S. pneumoniae immunization. (**A**) Serum IgG1 concentration; (**B**) Serum IgG2a concentration; (**C**) Ratio of IgG1/IgG2a; (**D**) Serum IL-4 concentration in ; (**E**) Relative expression of IL-4 mRNA on PND14; (**F**) Relative expression of IFN-γ mRNA on PND14; (**G**) Ratio of IL-4 and IFN-γ on PND14; (**H**) Relative expression of IL-4 mRNA on PND49; (**I**) Relative expression of IFN-γ mRNA on PND49; (**J**) Ratio of IL-4 and IFN-γ on PND49. Mean ± SD, n = 6–10. ^*^P < 0.05, ^**^P < 0.01 vs control.

In mice, Th1 immune responses produce IgG2a, and Th2 immune responses promote the production of IgG1. The ratio of IgG1/IgG2a is usually used to reflect the tendency of immune response. S. pneumoniae, belongs to T cell-dependent antigen, was used to induce the body to produce memory cells and IgG antibodies. The results showed that there were no changes in IgG1, IgG2a, the ratio of IgG1/IgG2a and IL-4 in serum before and after immunization with S. pneumoniae in control offspring (Fig. 2A–D). But in the PNE group, the levels of IgG2a were significantly decreased (P < 0.01, Fig. 2B), and levels of IgG1 and the ratio of IgG1/IgG2a were significantly increased (*P* < 0.01, Fig. 2A,C) when compared with the control after immunization. Moreover, the levels of IL-4 in serum of PNE male offspring were significantly higher than the controls both before and after immunization (*P* < 0.01, Fig. 2D).

#### The mRNA expression of IL-4 and interferon-γ (IFN-γ) in spleen on PND14 and PND49

As the specific cytokine for Th2 and Th1, the mRNA expression of IL-4 and IFN-γ we detected in spleen. On PND14, the levels of IL-4, IFN-γ mRNA expression and the ratio of IL-4/IFN-γ in PNE male offspring were significantly higher compared with the control (*P* < 0.01, *P* < 0.05, Fig. 2E–G). On PND49, the mRNA expression of IFN-γ was significantly decreased (*P* < 0.01, Fig. 2I), the mRNA expression of IL-4 and the ratio of IL-4/IFN-γ were significantly increased after immunization in PNE group (*P* < 0.01, Fig. 2H,J). However, there were no changes of the mRNA expression of IFN-γ and the ratio of IL-4/IFN-γ in the control after S. pneumoniae immunization (Fig. 2I,J).

#### Thymocyte phenotypes in offspring on PND14 and PND49

**Figure 3:**
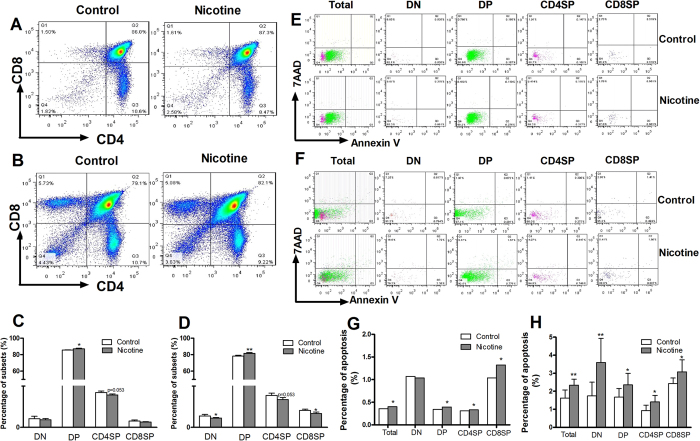
Effects of prenatal nicotine exposure (PNE) on the thymocyte phenotype and thymocyte apoptosis percentage in male offspring on PND14 and PND49. (**A**) Typical flow diagram of thymocyte phenotype on PND14; (**B**) Typical flow diagram of thymocyte phenotype on PND49; (**C**) Percentage of subsets on PND14; (**D**) Percentage of subsets on PND49. (**E**) Typical flow diagram of thymocyte apoptosis percentage on PND14; (**F**) Typical flow diagram of thymocyte apoptosis percentage on PND49; (**G**) Apoptosis percentage of thymocytes on PND14; (**H**) Apoptosis percentages of thymocytes on PND49. ^*^P < 0.05, ^**^P < 0.01 vs control.

Because the immune responses in peripheral depend on the effector T cells, which mainly originate from thymocytes. We examined the thymocyte phenotypes at PND14 and PND 49. On PND14, the percentage of DP in PNE male offspring was significantly increased (*P* < 0.05, Fig. 3A,C). And the percentage of CD4SP showed a decreased tendency (*P* = 0.053, Fig. 3A,C) in PNE group. On PND49, as compared with the control, the percentages of DN and CD8SP were significantly decreased, and the DP was significantly increased (*P* < 0.05, *P* < 0.01, Fig. 3B,D). Moreover, the CD4SP had a decreasing trend in PNE group (*P* = 0.053, Fig. 3B,D).

### Thymocyte apoptosis in male offspring on PND14 and PND49

Further, we detected thymocytes apoptosis to observe the correlation between apoptosis and phenotype. On PND14, the apoptosis percentages of total thymocytes, DP, CD4SP and CD8SP in PNE male offspring were higher than those in the controls (Fig. 3E,G). On PND49, the apoptosis percentages of total thymocytes and each phenotype in PNE male offspring were also increased compared with the controls (*P* < 0.01, *P* < 0.05, Fig. 3F,H).

## Results of fetus

### Developmental parameters of fetus

**Figure 4:**
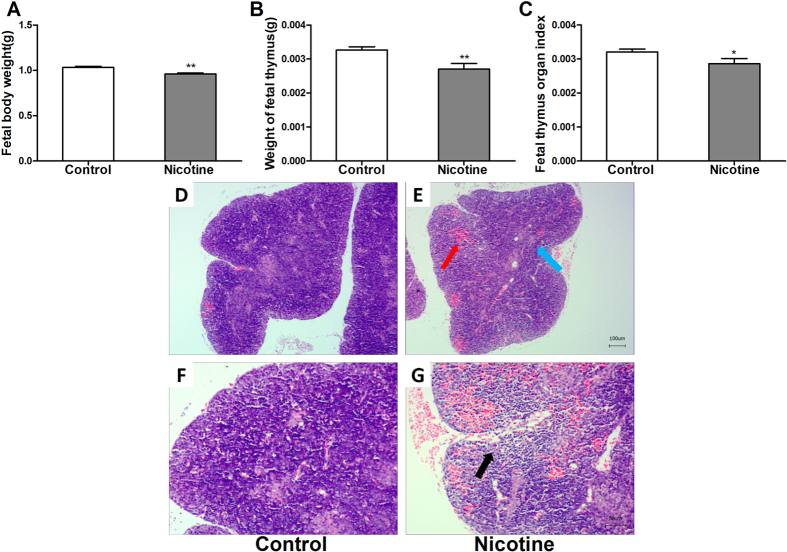
Effects of prenatal nicotine exposure (PNE) on the fetal body weight, fetal thymus weight, fetal thymus organ index and fetal thymus histological changes. (**A**) Fetal body weight; (**B**) Thymus weight of fetus; (**C**) Fetal thymus organ index; (**D**) control group (HE, ×40); (**E**) nicotine group (HE, ×40); (**F**) control group (HE, ×100); (**G**) nicotine group (HE, ×100). Mean ± SD, n = 8–10. ^*^P < 0.05, ^**^P < 0.01 vs control.

The fetal body weight, fetal thymus weight and fetal thymus organ index in PNE group were significantly lower than those in control (*P* < 0.01, *P* < 0.05, Fig. 4A–C).

### Histological changes in fetal thymus

Hematoxylin and eosin (HE) staining detection showed that there was obvious hemorrhage (red arrow) in PNE group, and the number of cells around the hemorrhage was decreased (black arrow) (Fig. 4E,G) compared with control (Fig. 4D,F). In addition, the boundary between cortex and medulla in PNE group is indistinct (blue arrow) (Fig. 4E).

### Thymocyte phenotypes in fetus

**Figure 5:**
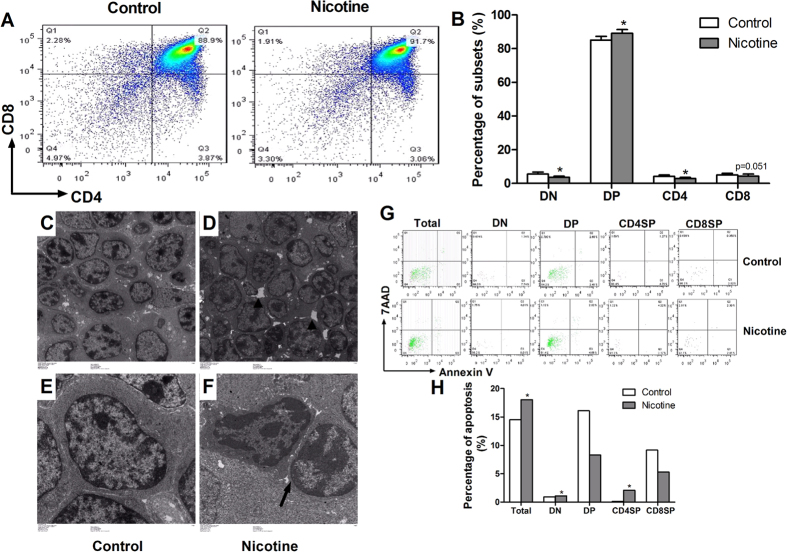
Effect of prenatal nicotine exposure (PNE) on the fetal thymocyte phenotypes and the fetal thymocyte apoptosis percentage. (**A**) Typical flow diagram of fetal thymocyte phenotypes; (**B**) Percentage of subsets in fetal mice; (**C**) control group (TEM, ×1000); (**D**) nicotine group (TEM, ×1000); (**E**) control group (TEM, ×3000); (**F**) nicotine group (TEM, ×3000); (**G**) Typical flow diagram of fetal thymocyte apoptosis percentage; (**H**) Apoptosis percentage of fetal thymocytes. ^*^P < 0.05, ^**^P < 0.01 vs control.

As compared with the control, the percentages of DN and CD4SP in PNE group were significantly lower, and DP was significantly higher (*P* < 0.05, Fig. 5A,B). Moreover, the percentage of CD8SP had a decreasing trend (*P *= 0.051, Fig. 5A,B).

### Thymocyte apoptosis in fetus

The transmission electron microscope (TEM) and flow cytometry were used to analyze the apoptosis of fetal thymocytes. The results of TEM showed that the intercellular spaces among fetal thymocytes were larger in PNE group (Fig. 5D) compared with the control (Fig. 5C). The apoptotic cells in nicotine group showed incomplete nuclear membrane and condensed chromatin (Fig. 5F) when compared with normal cells (Fig. 5E). Flow cytometry analysis indicated that the apoptosis percentages of total thymocytes, DN and CD4SP in PNE group were increased compared with the controls (Fig. 5G,H). The results of three times repetition were consistent with the above results.

### The mRNA expression of apoptosis signaling pathway in fetal thymus

**Figure 6:**
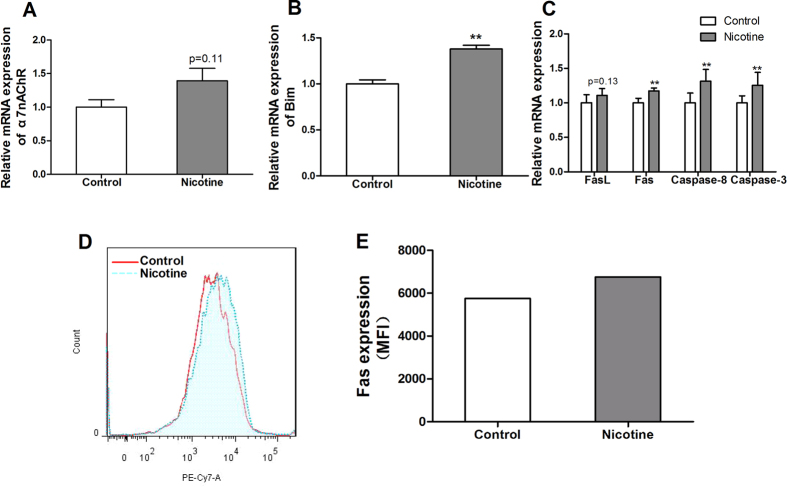
Effects of prenatal nicotine exposure (PNE) on the mRNA expression of nicotinic acetylcholine receptors α7 (α7 nAChR), Bcl-2 family member (Bim), genes about death receptor apoptosis pathway and the protein expression of Fas on the surface of fetal thymocytes. (**A**) Relative mRNA expression of α7 nAChR; (**B**) Relative mRNA expression of Bim; (**C**) Relative mRNA expression of death receptors apoptotic pathway; (**D**) Typical flow diagram of mean fluorescence intensity; (**E**) the protein expression of Fas on the surface of fetal thymocytes. FasL, Fas ligand. Mean ± SD, n = 6–10. ^*^P < 0.05, ^**^P < 0.01 vs control.

Further we detected the apoptosis pathway in fetal thymocytes. As compared with the control, the mRNA expression of α7 nAChR in nicotine group had an increasing trend (*P* = 0.11, Fig. 6A). As we observed the increased apoptosis percentage of CD4SP, we detected Bcl-2 family member (Bim), which was associated with negative selection of thymocytes. And the mRNA expression of Bim was significantly increased (*P* < 0.01, Fig. 6B). Furthermore, the mRNA expression of death receptor apoptosis pathway-related genes (Fas, FasL, caspase-8 and caspase-3) in PNE group were significantly higher than those in the controls (*P* < 0.01, Fig. 6C).

### The protein expression of Fas in fetal thymocytes

The data from flow cytometry analysis indicated that the mean fluorescence intensity of Fas in thymocytes of PNE fetus was higher than that in the control (Fig. 6D,E). The results represented in Fig. 6D,E were repeated three times.

## Discussion

It is known that active or passive smoking is a risk factor during pregnancy, which was associated with numerous short-term or long-term effects on the offspring^6^. In the present study, we established PNE mice models, 1.5 mg/kg of nicotine twice a day was chosen. Somm *et al*. reported that 3 mg/kg/d of prenatal nicotine exposure *via* osmotic minipump resulted in circulating nicotine metabolite levels in gestating rats are similar to those found in smokers consuming 10–19 cigarettes daily and to those reported in pregnant women using nicotine patches as tobacco substitutes^26^,^27^,^28^. In addition, another research showed administration of nicotine at the dose of 6 mg/kg/day produces plasma levels at the upper limit of those achieved in heavy smokers, whereas 2–3 mg/kg/day simulates moderate smoking^29^,^30^. According to the Guidelines on nicotine dose selection for in *vivo* research, mice are less sensitive to the effects of nicotine than the rats are, and therefore, require a higher nicotine dose to achieve a similar response^31^. Therefore, the dose of nicotine used in present study could equate to a moderate smoker. In mice, the blastosphere develops on GD 3.5, then embryo implantation is initiated on GD 4.5 until to GD 6.5. The fetal thymus develops from GD 11.5 to birth^32^. An *in vitro* research has showed that nicotine can reduce the hatched blastocyst and impair embryo development^33^. Moreover, an experiment on the effect of nicotine in different gestation period on offspring proved that the effect was more significant during the second and third trimester, especially the third trimester^34^,^35^. Therefore, the exposure period of nicotine from GD9 to GD18 in our study not only covered the critical period of fetal thymus development, but also minimized the influence of nicotine exposure on the embryo implantation. Results in the present study showed that PNE significantly decreased the body weight and the fetal thymus organ index. And histological changes in the fetal thymus was obvious in nicotine group. Therefore, nicotine, as one of the main harmful components of tobacco, could directly damage the development of fetal thymus.

Th1 and Th2 both belong to CD4^+^ T cells. Th1 produce IFN-γ as signature cytokine, which can promote the IgG2a antibody synthesis by B cells. Th2 are characterized by the cytokines IL-4, IL-5 and IL-13, which stimulate B cells to produce IgG1^36^. Th1 and Th2 are functionally balanced under normal conditions and the imbalance of Th1/Th2 is associated with immuno-functional disorders. The ratio of IgG1/IgG2a is usually used to reflect the tendency of immune response. A reduction of the ratio of IgG1/IgG2a is associated with a Th1 shift, while the enhancement of Th2 cellular response includes elevated ratio of IgG1/IgG2a. Researchers found that adverse intrauterine environment could change the ratio of IgG1/IgG2a. It was reported that exposure of mice to 5 or 50 ppm toluene during pregnancy resulted in increased IgG1 and decreased IgG2a in the plasma of infant mice (3 weeks old). The results suggested there was a Th2 skewing in the offspring^37^. In addition, maternal exposure to airborne particulate matter could cause postnatal immunological dysfunction in mice offspring, as evidenced by decreased total IgG2a and increased total IgG1 levels in the plasma^38^. S. pneumoniae used in this study belongs to T cell-dependent antigen, which can induce the body to produce memory cells and IgG antibodies. Our data demonstrated that the ratio of IgG1/IgG2a in PNE male offspring was increased after immunization. These results suggested that PNE could alter postnatal immune function in male offspring, resulting in a Th2 shift after antigen stimulation. Moreover, our data was consistent with the other results that prenatal secondhand cigarette smoke promoted Th2 polarization^39^.

Cytokines are involved in affecting or regulating most of the immune responses. Thus, we detected the mRNA expression of IL-4 (Th2-specific) and IFN-γ (Th1-specific) in spleen on PND14 and PND49, and the contents of IL-4 in serum on PND49 in this study. These results were expected to reflect the deviation of immune responses indirectly. Lots of studies demonstrated that adverse intrauterine environment could affect the production and expression of IL-4 and IFN-γ. It was reported that prenatal tetrachlorodibenzo-p-dioxin (TCDD) exposure could increase the IL-4 production of splenic immune cells in male offspring^40^. In addition, prenatal maternal stress could enhance the Th2 cytokines IL-4 and IL-13^41^. Furthermore, mice immunized with OVA mounted strong Th2 responses as indicated by the significant level of IL-4 and undetectable level of IFN-γ^42^. In present study, the results of real-time quantitative PCR (RT-qPCR) showed that the ratio of IL-4/IFN-γ in PNE male offspring was higher than that of control on PND14 and PND49. These results suggested that PNE could cause persistent changes in the immune response in male offspring, from early life to adolescence, which manifested as a Th2 shift. Our pre-experiments showed that the concentrations of IFN-γ in serum before and after immunization are both below the detection limit of the enzyme linked immunosorbent assay (ELISA) kits (5 pg/mL). Thus, we only detected the IL-4 in serum. The increased content of IL-4 in serum in PNE group after immunization further confirmed that PNE could enhance Th2-mediated immune responses.

Immune responses in peripheral depend on the effector T cells (such as Th), which mainly originate from thymocytes. In the embryo, the differentiation of thymocytes can be divided into four stages. Many studies indicated that adverse intrauterine environment could change the percentage or ratio of the thymocytes. It was reported that prenatal cadmium exposure could increase the percentage of CD4SP and the ratio of CD4/CD8 in fetal thymus^43^. Prenatal TCDD exposure could reduce the number of DP in fetal thymus^44^. In addition, the effects of adverse intrauterine environment on fetal thymocytes could last until after birth. Researchers found that carbon black nanoparticle exposure during middle and late pregnancy could increase DN and DP at different time points^19^. In this study, we also examined the thymocyte phenotypes at three time points, which were gestational day (GD)18, PND14 and PND 49, respectively. We found that the variation of phenotypes with age in PNE offspring were consistent with those of controls (Supplementary Figure S1). Moreover, there were similar changing trends of thymocytes in nicotine group compared with the control at three time points. These results suggested that PNE might have programming effects on the thymocytes.

Th are derived from naïve CD4^+^ T cells. Researchers found the proportion of Th1 increased progressively with age in mice. However, the percentage of Th2 decreased during the first three weeks of postnatal life^45^. These results were consistent with human or primate subjects^46^,^47^. That is, Th2 is at a high level in newborn, but Th1 is at a low level under normal circumstances. Wang *et al*.^45^ found that prenatal particulates exposure could lead to a reduction of CD4^+^ T cells. As the mice matured, Th2 recovered and eventually became equivalent to control mice. While Th1 remained suppressed even at 6 weeks of age compared with the controls^45^. Thus, the reduction of CD4^+^ T cells might trigger a shift of Th2. The failure to increase Th1 cell numbers might predispose the individual to Th2 dominated immune responses following allergen exposure. In this study, the proportion of CD4SP was significantly reduced in PNE group, and the decreasing trend was last to PND49. We speculated this might be the reason for the skewing of Th2 in male offspring.

In embryo, nearly 95% immature T cells undergo apoptosis and only a little can develop into naïve T cells after migrating into thymus. Apoptosis is crucial during the development process of thymocytes. Increased apoptosis of thymocytes in fetal stage can reduce the volume and weight of thymus, also can change the phenotypes of thymocytes, all of which often result in thymus dysfunction after birth. Studies showed that adverse intrauterine environment might increase the apoptosis percentage of fetal thymocytes, thus affected the thymus function after birth. It was reported that prenatal TCDD could promote the thymocyte apoptosis and finally resulted in thymus atrophy in offspring^48^. In addition, prenatal betamethasone exposure could induce the apoptosis of DP, and reduce the proliferation of immature thymocytes^49^. In our study, the results of both TEM and flow cytometry showed that the total apoptosis rate of fetal thymocyte in nicotine group was increased. Moreover, the increased mRNA expression of α7 nAChR and genes about death receptor-mediated apoptotic pathway and the protein expression of Fas in fetal thymocytes further indicated that PNE could increase the total apoptosis rate of fetal thymocyte through α7 nAChR/Fas-mediated apoptotic pathway.

Due to the crucial role of apoptosis in every developmental stage of thymocytes, we also analyzed the apoptosis percentages of thymocytes in different stages. Results in the present study showed that the apoptosis percentages of fetal DN and CD4SP in nicotine group were higher than those of control. Moreover, the increased apoptosis percentage of CD4SP also occurred in PNE offspring on PND14 and PND49. The reduced cell proportion of CD4SP was consist with the increased apoptosis at the three time points. These results suggested that PNE could decrease CD4SP through increasing its apoptosis percentages, which could last to postnatal early life, even to adolescence. It is known that incompetent SP mainly underwent negative selection and occurred apoptosis before leaving thymus. It was reported that the negative selection of thymocytes was mediated by Ca^2+^-dependent transcriptional induction of Bim^50^. Studies showed that the effects of nicotine on thymocytes occurred through the interaction with α7 nAChR, which can increase the intracellular levels of Ca^2+ ^51. Thus, we speculated that nicotine might promote the negative selection of SP to reduce the CD4SP in thymus. In this study, the expression of α7 nAChR on fetal thymocytes in nicotine group had an increased trend, and Bim was significantly upregulated. These results suggested that PNE could increase CD4SP negative selection mediated by Bim and induce its apoptosis. In summary, PNE not only increased the apoptosis of total fetal thymocytes through Fas-mediated apoptotic pathway, but also promoted the negative selection of CD4SP. Both of them caused the reduced cell proportion of CD4SP and output of CD4^+^ naïve T cells.

For adult mice, different mouse strains may display different immune responses. For example, BL/6 mice showed a predominant Th1-like immune responses whereas BALB/c mice displayed predominant Th2 responses. In the mechanism study, we aimed to clarify the developmental origin mechanism of the effects of prenatal nicotine exposure from the point of thymocytes apoptosis. The development process of thymocytes and the apoptotic pathways in thymocytes were consistent in different mouse strains. Moreover, it was reported that gestational exposure of BALB/c and C57BL/6 mice to secondhand cigarette smoke both could affect the development of fetuses, and the influences were consistent^52^. Therefore, we speculated that the influences of nicotine on the development of fetal thymocyte may be similar between BALB/c and C57BL/6 mice, from the perspective of developmental toxicology.

## Conclusion

**Figure 7:**
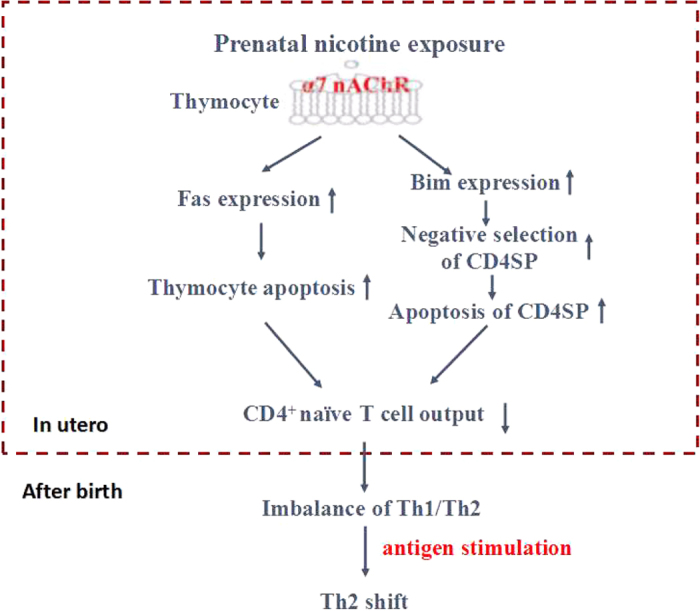
The mechanism hypothesis for the reduced CD4 naïve T cell output and Th2 shift in male offspring induced by prenatal nicotine exposure (PNE). α7 nAChR, nicotinic acetylcholine receptors α7; Bim, Bcl-2 family member; Th, T helper cells.

Taken together, PNE has direct toxic effects on the development of fetal thymocytes, resulting in immune dysfunction in offspring through triggering a Th2 shift. The underlying mechanism (Fig. 7) may be that PNE decreases the output of CD4^+^naïve T cells through increasing the apoptosis of total thymocytes and CD4SP. On the one hand, nicotine interacted with α7 nAChR, and subsequently activated Fas-mediated apoptotic pathway to increase the apoptosis of fetal thymocytes. On the other hand, nicotine increased the negative selection of CD4SP induced by Bim. These two pathways lead to the reduced output of CD4^+^naïve T cells, which is the reason for the skewing of Th2 after antigen stimulation in male offspring. Our present study provides an underlying mechanism of developmental origin of immune dysfunction due to PNE.

## Materials and Methods

### Chemicals and reagents

Nicotine was purchased from Sigma-Aldrich (St. Louis, MO, USA). Monoclonal antibodies (anti-mouse CD3-FITC, anti-mouse CD4-APC, anti-mouse CD8-PE-cy7, Rat IgG2b K Isotype Control FITC, Rat IgG2b K Isotype Control APC and Rat IgG2a K Isotype Control PE-cy7) and Annexin V PE Apoptosis Detection kit were purchased from eBioscience (San Diego, USA). Anti-mouse CD95-PE-cy7 was obtained from BD Biosciences (New Jersey, USA). Mouse IgG1 and IgG2a ELISA kits were obtained from MultiSciences (Hangzhou, Zhejiang, China). Mouse IL-4 ELISA kits were obtained from Dakewe Biotech (Shenzhen, Guangdong, China). Trizol was purchased from Life Technologies (Gaithersburg, MD, USA). Reverse transcription and RT-qPCR kits were purchased from TaKaRa Biotechnology (Dalian, Liaoning, China). All primers were synthesized by Sangon Biotech Co., Ltd. (Shanghai, China). All chemicals and reagents were analytical grade.

### Animals and treatment

Specific pathogen-free nulliparous female (22 ± 2 g) and male (25 ± 2 g) Balb/C mice were obtained from the Experimental Center of Hubei Medical Scientific Academy (No. 2008-0005, Wuhan, Hubei, China). The animal experiments in this study were performed in the Center for Animal Experiment of Wuhan University (Wuhan, Hubei, China), which has been accredited by the Association for Assessment and Accreditation of Laboratory Animal Care International (AAALAC International). The protocol was approved by the Committee on the Ethics of Animal Experiments at the Wuhan University School of Medicine (Permit Number: 14016). All animal experiment procedures were performed in accordance with the Guidelines for the Care and Use of Laboratory Animals of the Chinese Animal Welfare Committee and the International Council on Research Animal Care.

**Figure 8:**
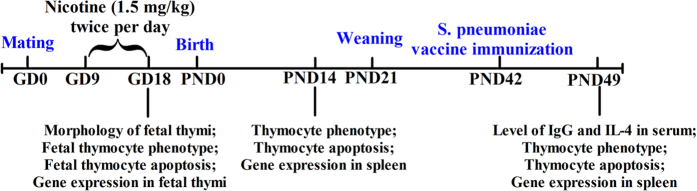
The schedule of animal treatment from gestational day 0 (GD0) to postnatal day 49 (PND49).

Mice were maintained under controlled conditions of temperature (22–24 °C), humidity (45–65%), and light (a 12 h light-dark cycle), with free access to food and water. Animals were allowed to acclimate for at least one week before subjected to experimental conditions. A schematic of the procedure for maternal and offspring mouse treatment was shown in Fig. 8. Female mice mated with male mice in 2:1 overnight. The day was declared as GD 0 if a vaginal plug was found in the vagina. The pregnant mice were randomly divided into two groups: a nicotine group and a control group. From GD9 to GD18, the mice in nicotine group were subcutaneously administered 1.5 mg/kg of nicotine twice per day. The control mice were treated with the same volume of the vehicle (saline). At GD18, some pregnant mice were sacrificed under isoflurane anesthesia, and their fetuses were removed quickly from the uterus and weighed. Fetal thymi from each littermate were collected and pooled into one sample, and 3 pooled samples were used for flow cytometry. In addition, 3 fetal thymi were randomly selected for fixing in phosphate-buffered 10% neutral formalin solution or 2.5% glutaraldehyde solution for HE staining and TEM analysis. The remaining fetal thymi of the littermates were pooled together as one sample, immediately frozen in liquid nitrogen, and stored at −80 °C for subsequent experiments.

For the experiment with offspring mice, pregnant mice were kept until normal delivery (GD19), and on PND0 the numbers of pups were normalized to 7 pups per litter to assure adequate and standardized nutrition. On PND14, one male pup per litter was selected randomly from 8 different mothers in each group, and euthanized. The thymi and spleens were removed for flow cytometry and gene expression detection respectively. Other pups were normal fed until weaning (PND21). During the first week after birth, the offspring might be eaten by the mothers because of the changed smell after we touch them. Therefore, we didn’t number and weigh the offspring in the first week. The offspring were numbered on PND7 to avoid being eaten. Bodyweights of the offspring were measured weekly, and the corresponding bodyweight gain rate was calculated as follows: Gain rate in body weight (%) = [(body weight of PND × −body weight of PND7)]/body weight of PND7 × 100%. On PND42, half of the male offspring were immunized with a S. pneumoniae vaccine for 1 week and were sacrificed for tissue collection after anesthesia on PND49. Thymi of 3 offspring from each group were harvested for flow cytometry. Serum was prepared and stored at −80 °C for analysis. The spleens were immediately frozen in liquid nitrogen, and then stored at −80 °C for subsequent experiments.

### Cell preparation, staining and flow cytometry

Thymi from fetuses and offspring were harvested and placed in flow cytometry staining buffer. Single cell suspensions were prepared as follow. The thymus was gently dissociated over a stainless steel sieve screen using a plunger of 2 mL syringe. Cells were washed twice in staining buffer for 5 min, 500 × g and 4 °C. The supernatant was discarded and the cell pellet was resuspended in staining buffer. Viable cells were enumerated using trypan blue and a hemacytometer. Cells were adjusted to 1 × 10^7^ cells/mL. The cell preparations from each litter or pup were analyzed individually. Thymocytes were stained with the fluorochrome directly conjugated antibodies (anti-mouse CD3-FITC, anti-mouse CD4-APC, anti-mouse CD8-PE-cy7 or anti-mouse CD95-PE-cy7 only). Anti-CD3, anti-CD4 and anti-CD8 were used to identify DN, DP and SP cell subpopulations. To analyze the expression of Fas, CD95 expression was detected. The cells were stained for 30 min in the dark at 4 °C, washed twice and resuspended with staining buffer. For apoptosis analysis, the Annexin V Apoptosis Detection kit was used. The cells surface staining was performed as described above followed by being washed, resuspended in Binding Buffer at 10^6^ cells/mL. Then the cells were incubated 10 min with Annexin V at the room temperature and protected from light. After incubation, the cells were washed, and resuspended in 200 μL Binding Buffer. 7-Aminoactinomycin D (7-AAD) was added before being analyzed by flow cytometry. Data were acquired on a BD FACSAria^TM^ III (BD Biosciences) using FASCDiva Software at the Flow Cytometry Facility of Wuhan University. All analysis was performed using FlowJo software (Tree Star Inc.). The detection was repeated three times.

### Histological examination

For HE staining, the fetal thymus samples were fixed in 10% formaldehyde, then embedded in paraffin, sliced at 3 μm, and stained with HE for light microscopic examination (100×, Olympus BX-53). For TEM, the fetal thymus samples were excised into small pieces (<1 mm^3^) and fixed overnight in 2.5% glutaraldehyde in phosphate buffer. Samples were then washed in 0.1 M sodium cacodylate buffer and postfixed with 1% osmium tetroxide. Samples were dehydrated through a graded series of ethanol and embedded in EPON812. Ultrathin sections (70 nm thick) were obtained with an EM UC7 ultramicrotome (Leica, Germany), dually stained with uranyl acetate and lead citrate, and examined with a HT-7700 transmission electron microscope (Hitachi, Tokyo, Japan).

### Levels of total immunoglobulin G1 (IgG1), IgG2a antibodies and IL-4 in serum in offspring on PND49

Serum samples were balanced to room temperature. Total IgG1, IgG2a antibodies, IL-4 in serum in offspring were measured using the ELISA kits according to the manufacture’s protocols. The dilution ratio of serum samples for IgG1 detection was 1:10000, and for IgG2a was 1:2000, while there was no dilution for IL-4 detection.

### RNA preparation and qPCR

Total RNA were isolated from the spleen and fetal thymus using Trizol reagent, according to the manufacturer’s protocol. The concentration and purity of the isolated RNA were determined by a spectrophotometer, and the concentration of each sample was adjusted to 1 μg/μL. Single-strand cDNA was prepared using the reverse transcription kit. All the primer sequences shown in Supplementary Table S1 were designed by Primer Premier 5.0 from PREMIER Biosoft International (Palo Alto, CA, USA) and queried by NCBI BLAST database for homology comparison. PCR assays were performed using a QuantStudio 6 Flex from Applied Biosystems (Foster City, CA, USA) in a total volume of 20 μl reaction mixture containing 1 μl of cDNA template, 0.4 μl of 10 μM each primer, 10 μl of 2 × Premix Ex Taq, 0.4 μl ROX and 7.8 μl of DEPC-H_2_O. PCR cycling conditions were as follows: 30 s at 95 °C for pre-denaturation, 5 s at 95 °C for denaturation, and appropriate annealing conditions for each gene (listed in Supplementary Table S1). Each gene was calculated by ΔΔCt method using the ribosomal housekeeping gene glyceraldehyde phosphate dehydrogenase (GAPDH) as internal controls.

### Statistical analyses

SPSS 17 (SPSS Science Inc., Chicago, Illinois) and Prism (GraphPad Software, Inc., La Jolla, CA, USA, version 5.0) were used for data analysis. Results were reported as means ± standard deviation (SD). All measurement data were evaluated with Student’s t test or with two-factor analysis of variance as appropriate. Statistical significance was set at *P* < 0.05.

## Additional Information

**How to cite this article**: Chen, T. *et al*. Increased Fetal Thymocytes Apoptosis Contributes to Prenatal Nicotine Exposure-induced Th1/Th2 Imbalance in Male Offspring Mice. *Sci. Rep.* **6**, 39013; doi: 10.1038/srep39013 (2016).

**Publisher's note:** Springer Nature remains neutral with regard to jurisdictional claims in published maps and institutional affiliations.

## Supplementary Material

Supplementary Information
