# Supplementary material for: Increased Fetal Thymocytes Apoptosis Contributes to Prenatal Nicotine Exposure-induced Th1/Th2 Imbalance in Male Offspring Mice
Source: Sci Rep. 2016 Dec 15;6:39013. doi: 10.1038/srep39013 (PMC5157046; doi:10.1038/srep39013)
Supplement: Supplementary Information [file srep39013-s1.pdf]

Title page

Increased Fetal Thymocytes Apoptosis Contributes to Prenatal Nicotine Exposure-induced Th1/Th2 Imbalance in Male Offspring Mice

Ting Chen[+], You-e Yan[+], Sha Liu, Han-xiao Liu, Hui-yi Yan, Li-fang Hou, Wen Qu & Jie Ping[*]

Department of Pharmacology, Wuhan University School of Basic Medical Sciences, Wuhan 430071, China.

***Corresponding author: Jie Ping**

Address: 185, Donghu Road, Wuhan 430071, China.

Tel.: +86 27 6875 8665.

Fax: +86 27 8733 1670.

E-mail: pingjie@whu.edu.cn

[+]These authors contributed equally to this work.

Supplementary Figure S1

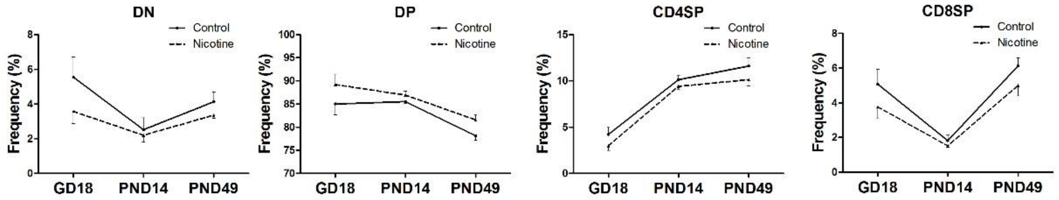

Supplementary Figure S1. **The thymocyte phenotypes in male offspring at different time points.** DN, double negative cell; DP, double positive cell; SP, single positive cell; GD, gestational day; PND, postnatal day.

Supplementary Figure S2

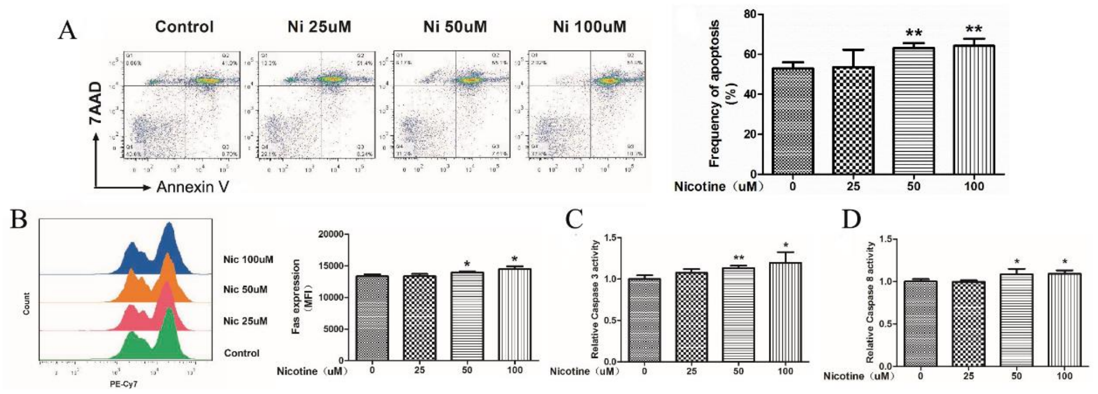

Supplementary Figure S2. **Effects of nicotine (25, 50 and 100 μM) on cell apoptosis, Fas expression, caspase 3 and caspase 8 activity in primary thymus cells.** Thymus cells were prepared from weaned BALB/c mice and cultured with different concentrations of nicotine for 48 h. A. Cell apoptosis was detected by flow cytometry; B. Protein expression of Fas on the surface of thymus cells; C and D: caspase 3 and caspase 8 activity in thymus cells. Mean ± SD, n=6. **P*<0.05, ***P*<0.01 *vs* control.

Supplementary Table 1

Supplementary Table 1. Oligonucleotide primers and PCR conditions of mouse in quantitative real-time PCR.

Genes	Forward primer	Reverse primer	Product （bp）	Annealing
α7nAChR	CACATTCCACACCAACGTCTT	AAAAGGGAACCAGCGTACATC	106	60°C,30 s
Bim	CCGGAGATACGGATTGCACAG	CAGCCTCGCGGTAATCATTTG	97	60°C,30 s
Caspase-3	CGTGGTTCATCCAGTCCCTTT	ATTCCGTTGCCACCTTCCT	102	60°C,30 s
Caspase-8	AGGTACTCGGCCACAGGTTA	TGGGATGTAGTCCAAGCACA	137	60°C,30 s
Fas	ATGCACACTCTGCGATGAAG	CAGTGTTCACAGCCAGGAGA	120	60°C,30 s
FasL	GCAGAAGGAACTGGCAGAAC	TTAAATGGGCCACACTCCTC	128	60°C,30 s
GAPDH	AACTTTGGCATTGTGGAAGG	GGATGCAGGGATGATGTTCT	132	60°C,30 s
IFN-γ	CTCAAGTGGCATAGATGTGGAAG	GATGGCCTGATTGTCTTTCAAG	120	60°C,30 s
IL-4	ATGGATGTGCCAAACGTCCT	AAGCACCTTGGAAGCCCTAC	78	58°C,30 s;
